# Supplementary material for: Voltage imaging reveals the dynamic electrical signatures of human breast cancer cells
Source: Commun Biol. 2022 Nov 11;5:1178. doi: 10.1038/s42003-022-04077-2 (PMC9652252; doi:10.1038/s42003-022-04077-2)
Supplement: Supplementary file 2 — Supplemental Material [file 42003_2022_4077_MOESM2_ESM.pdf]

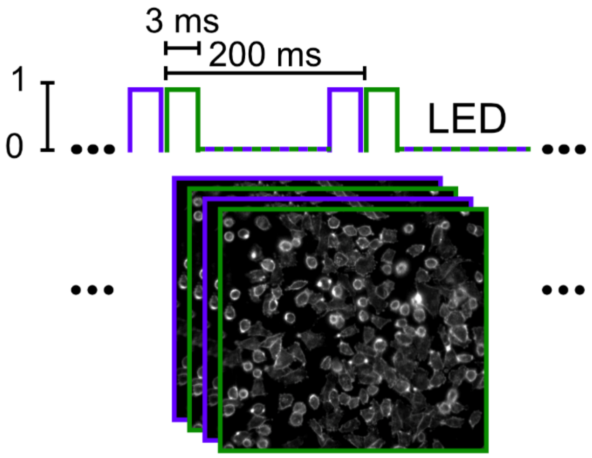

Figure S1. Image acquisition timing diagram. Top, illustration of the command signals sent to the LED illumination driver showing the length and duty cycle of the ratiometric illumination. Each color was sequentially strobed for 3 ms every 200 ms resulting in an interleaved series of fluorescent images (bottom).

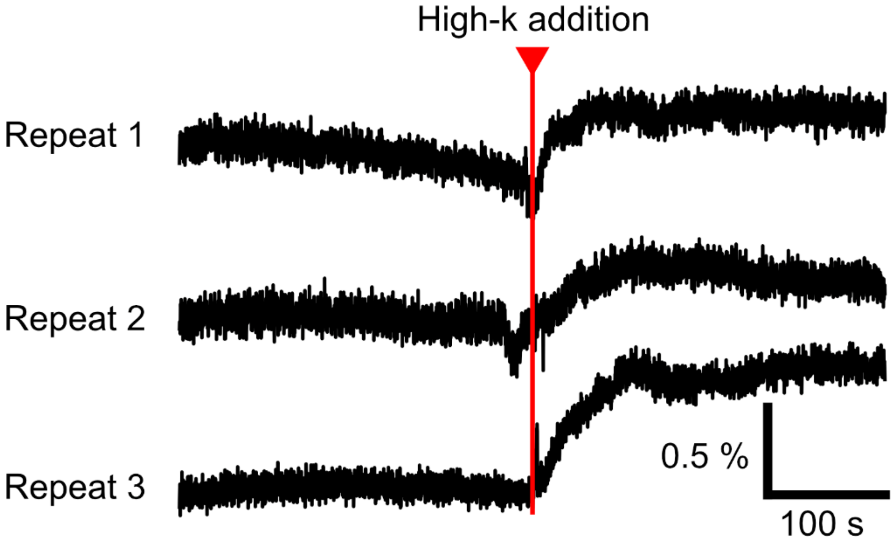

Figure S2. Perfusion of cells with a high potassium solution results in global depolarisation. Each trace shows the mean time course of all cells in the FOV during the perfusion of high potassium (containing 100 mM KCl) mammalian physiological saline. The resulting membrane depolarisation can be clearly observed in the voltage dye signal.

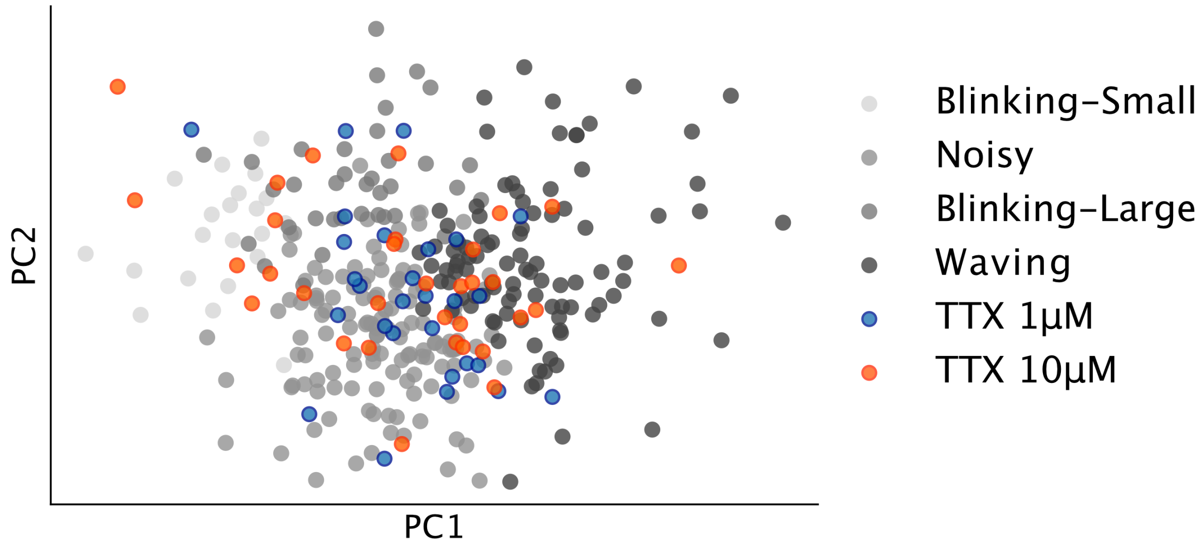

Figure S3. Treatment of MDA-MB-231 cells with TTX did not bias their Vm fluctuations with respect to the four DES classes. The projection of TTX-treated MDA-MB-231 cells (blue: 1 μM; orange: 10 μM) onto the untreated cells' 2D PC space shows distribution throughout the four DES classes (grey).
