# Supplementary material for: Voltage imaging reveals the dynamic electrical signatures of human breast cancer cells
Source: Commun Biol. 2022 Nov 11;5:1178. doi: 10.1038/s42003-022-04077-2 (PMC9652252; doi:10.1038/s42003-022-04077-2)
Supplement: Supplementary file 3 — Description of Additional Supplementary Files [file 42003_2022_4077_MOESM3_ESM.pdf]

Description of Additional Supplementary Files

File name: Supplementary Movie 1
Description: Exemplar video of voltage transients in MDA-MB-231 cells. Left, raw image of the blue fluorescent channel showing the voltage dye-stained cells. Right, video of the ratiometric pixel-wise ΔR/R0 calculated using the process described in the Methods. The video is shown at 25 frames/s (5x real speed). Darker colours correspond to hyperpolarisations and lighter colours to depolariastions. Large and short hyperpolarisations are the most prominent transients, and most cells display little or no voltage activity. This video has been spatially and temporally filtered with a gaussian of width (3,2,2) samples in (t,y,x) and the 0.5 and 99.5% percentile pixels are saturated to facilitate transient visualisation.

File name: Supplementary Movie 2
Description: Video of voltage transient wave. Left, raw image of the blue channel fluorescence. Right, video of the ratiometric pixel-wise ΔR/R0 calculated using the process described in the methods. A wave of negative transients can be seen propagating from cells in the lower right corner to the upper left corner at an approximate speed of 27 µm/s. The video is shown at 25 frames/s (5x real speed). Darker colours correspond to hyperpolarisations and lighter colours to depolarisations. This video has been spatio-temporally filtered with a gaussian of width (3,2,2) samples in (t,y,x) and the 0.5 and 99.5% percentile pixels are saturated to facilitate transient visualisation.

File name: Supplementary Data
Description: The source data behind the graphs in the paper
