# Supplementary material for: Voltage imaging reveals the dynamic electrical signatures of human breast cancer cells
Source: Commun Biol. 2022 Nov 11;5:1178. doi: 10.1038/s42003-022-04077-2 (PMC9652252; doi:10.1038/s42003-022-04077-2)
Supplement: Supplementary file 6 — Reporting Summary [file 42003_2022_4077_MOESM6_ESM.pdf]

nature portfolio

Corresponding author(s): Amanda Foust, Chris Bakal

Last updated by author(s): July 4, 2022

Reporting Summary

Nature Portfolio wishes to improve the reproducibility of the work that we publish. This form provides structure for consistency and transparency in reporting. For further information on Nature Portfolio policies, see our Editorial Policies and the Editorial Policy Checklist.

Statistics

For all statistical analyses, confirm that the following items are present in the figure legend, table legend, main text, or Methods section.

n/a	Confirmed	
☐	☒	The exact sample size (*n*) for each experimental group/condition, given as a discrete number and unit of measurement
☐	☒	A statement on whether measurements were taken from distinct samples or whether the same sample was measured repeatedly
☐	☒	The statistical test(s) used AND whether they are one- or two-sided<br>*Only common tests should be described solely by name; describe more complex techniques in the Methods section.*
☒	☐	A description of all covariates tested
☒	☐	A description of any assumptions or corrections, such as tests of normality and adjustment for multiple comparisons
☐	☒	A full description of the statistical parameters including central tendency (e.g. means) or other basic estimates (e.g. regression coefficient) AND variation (e.g. standard deviation) or associated estimates of uncertainty (e.g. confidence intervals)
☐	☒	For null hypothesis testing, the test statistic (e.g. *F*, *t*, *r*) with confidence intervals, effect sizes, degrees of freedom and *P* value noted<br>*Give P values as exact values whenever suitable.*
☒	☐	For Bayesian analysis, information on the choice of priors and Markov chain Monte Carlo settings
☒	☐	For hierarchical and complex designs, identification of the appropriate level for tests and full reporting of outcomes
☒	☐	Estimates of effect sizes (e.g. Cohen's *d*, Pearson's *r*), indicating how they were calculated

Our web collection on statistics for biologists contains articles on many of the points above.

Software and code

Policy information about availability of computer code

Data collection	Images were acquired in Micromanager 2. Electrophysiology data were acquired with Spike9 software (Cambridge Electronic Design).
Data analysis	All data analysis was performed in Python 3 using Numpy, SciPy, Tifffile, Scikit-image, Scikit-learn and Pandas. Figures were generated using MatPlotLib. The analysis code is available at https://github.com/peq10/cancer_vsd.

For manuscripts utilizing custom algorithms or software that are central to the research but not yet described in published literature, software must be made available to editors and reviewers. We strongly encourage code deposition in a community repository (e.g. GitHub). See the Nature Portfolio guidelines for submitting code & software for further information.

Data

Policy information about availability of data

All manuscripts must include a data availability statement. This statement should provide the following information, where applicable:

- Accession codes, unique identifiers, or web links for publicly available datasets
- A description of any restrictions on data availability
- For clinical datasets or third party data, please ensure that the statement adheres to our policy

The imaging and electrophysiological datasets generated and analysed during the current study are available from the corresponding author on reasonable request.

Field-specific reporting

Please select the one below that is the best fit for your research. If you are not sure, read the appropriate sections before making your selection.

☒ Life sciences ☐ Behavioural & social sciences ☐ Ecological, evolutionary & environmental sciences

For a reference copy of the document with all sections, see nature.com/documents/nr-reporting-summary-flat.pdf

Life sciences study design

All studies must disclose on these points even when the disclosure is negative.

Sample size	No sample sizes were pre-determined as this was an exploratory, observational study.
Data exclusions	Data were excluded at 3 stages:<br>1) Complete recordings were excluded when they failed or were too poor quality for analysis<br>2) Sections of optical Vm traces containing spurious signals due to transient occlusion during imaging by debris were excluded automatically during event segmentations as described in the methods.<br>3) After event segmentation individual cells were rejected if the segmentation was false due to imaging debris.
Replication	Experiments were repeated by recording from multiple locations per coverslip of cultured cells (technical replicate) and from multiple technical replicates per experiment. Exact numbers are given in the text.
Randomization	No explicit randomisation was used as it is not applicable to these experiments.
Blinding	No blinding was performed.

Reporting for specific materials, systems and methods

We require information from authors about some types of materials, experimental systems and methods used in many studies. Here, indicate whether each material, system or method listed is relevant to your study. If you are not sure if a list item applies to your research, read the appropriate section before selecting a response.

Materials & experimental systems

n/a	Involved in the study	
☒	☐	Antibodies
☐	☒	Eukaryotic cell lines
☒	☐	Palaeontology and archaeology
☒	☐	Animals and other organisms
☒	☐	Human research participants
☒	☐	Clinical data
☒	☐	Dual use research of concern

Methods

n/a	Involved in the study	
☒	☐	ChIP-seq
☒	☐	Flow cytometry
☒	☐	MRI-based neuroimaging

Eukaryotic cell lines

Policy information about cell lines

Cell line source(s)	MCF10-A cells were obtained from ATCC<br>MDA-MB-231 were a kind gift from Dr. Janine Erler<br>MDA-MB-468 were a kind gift from the Poulogiannis lab (Dr. George Poulogiannis) (ICR)<br>MDA-MB-453 were a kind gift from the Isacke lab (Professor Claire Isacke) (ICR)<br>SUM159 and hs578t were a kind gift from Dr. Rachel Natrajan (ICR)<br>T47D, BT474 and Cal51 were a kind gift from the Turner lab (Professor Nicholas Turner) (ICR)
Authentication	Cell lines were not authenticated by ourselves.
Mycoplasma contamination	Cell lines were regularly tested and confirmed to be mycoplasma-negative by PCR (e-Myco Mycoplasma PCRDetection Kit, iNtRON Biotechnology).
Commonly misidentified lines<br>(See ICLAC register)	None
